# Double-Negative Prostate Cancer Masquerading as a Squamous Cancer of Unknown Primary: A Clinicopathologic and Genomic Sequencing-Based Case Study

**DOI:** 10.1200/PO.20.00309

**Published:** 2020-11-16

**Authors:** Frank C. Cackowski, Chandan Kumar-Sinha, Rohit Mehra, Yi-Mi Wu, Dan R. Robinson, Joshi J. Alumkal, Arul M. Chinnaiyan

**Affiliations:** ^1^ Department of Medicine, Division of Hematology & Oncology, and Rogel Cancer Center, University of Michigan School of Medicine, Ann Arbor, MI; ^2^ Department of Oncology, Wayne State University School of Medicine and Karmanos Cancer Institute, Detroit, MI; ^3^ Michigan Center for Translational Pathology, University of Michigan School of Medicine, Ann Arbor, MI; ^4^ Department of Pathology and Rogel Cancer Center, University of Michigan School of Medicine, Ann Arbor, MI; ^5^ Michigan Center for Translational Pathology, Department of Computational Medicine and Bioinformatics, Howard Hughes Medical Institute, Department of Urology, and Rogel Cancer Center, University of Michigan School of Medicine, Ann Arbor, MI

## Case Description

**FIG 1.**
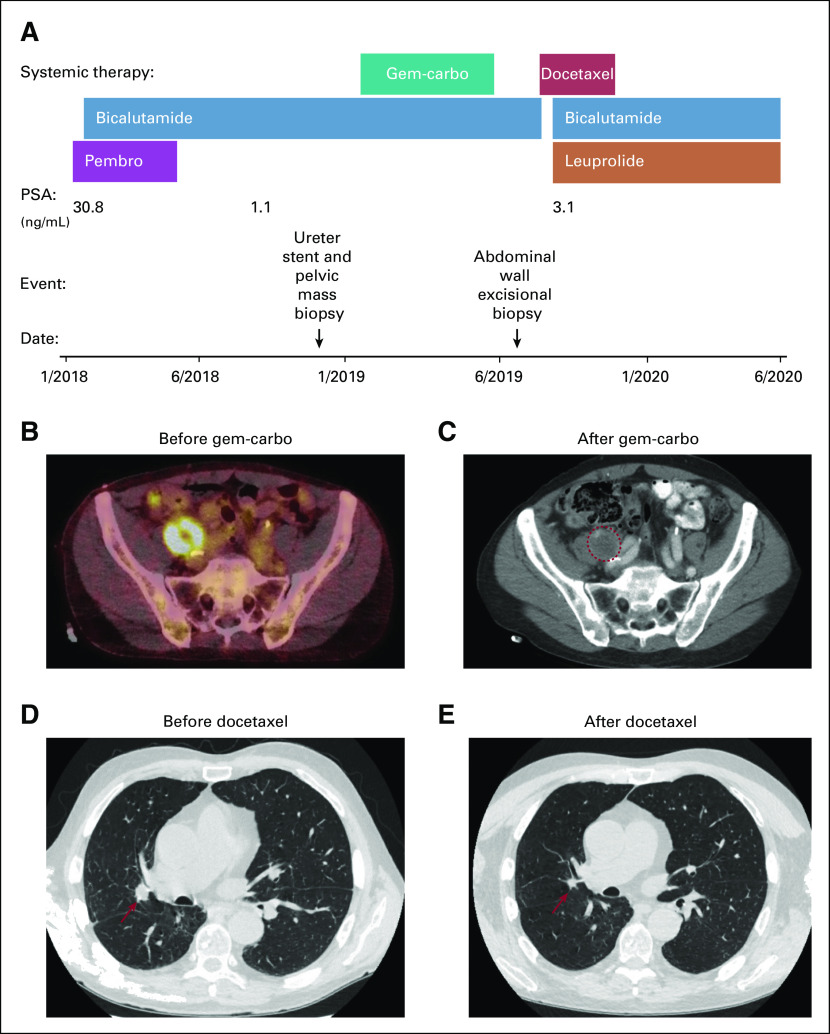
Clinical timeline and cross-sectional imaging. (A) Summary of the clinical course and systemic therapy. (B) Positron emission tomography (PET)/computed tomography (CT) showing the right pelvic mass before treatment in December 2018. Dark orange/white color indicates metabolic activity. (C) CT with oral and intravenous contrast showing the right pelvic mass after treatment with carboplatin and gemcitabine (Gem-carbo) chemotherapy. Dashed red circle indicates the metabolically active lesion in the previous PET scan. (D) Noncontrast chest CT before docetaxel chemotherapy in September 2019. (E) Noncontrast chest CT after three cycles of docetaxel chemotherapy. Red arrows in (D) and (E) indicate a lesion responsive to docetaxel. PSA, prostate-specific antigen.

In January 2018, a 79-year-old man was referred to our genitourinary medical oncology clinic for management of his prostate adenocarcinoma metastatic to multiple bones. His case was complicated by a concurrent diagnosis of melanoma metastatic to a distant skin site, for which he had started pembrolizumab immunotherapy the week previously. His prostate cancer had originally been diagnosed in November 2011 after a transurethral resection of the prostate performed for urinary obstruction revealed Gleason score 7 prostate adenocarcinoma. In June 2015, he was found to have biopsy-proven prostate adenocarcinoma from a rib metastasis. The patient elected to defer all medical therapy and had his rib metastasis treated with radiation alone. He then had definitive radiation to the prostate in June 2016. His prostate cancer spread to additional bony sites, which were likewise treated with radiation, including radiation to the thoracic spine in July 2017 and the proximal humerus in August 2017, rather than any systemic therapy. In December 2017, he had prophylactic nipple irradiation to prevent gynecomastia in preparation for noncastrating medical therapy with single-agent bicalutamide. On meeting the patient for the first time in January 2018, we elected to continue with the plan for single-agent bicalutamide 50 mg per day, given the patient’s preference and the uncertain prognosis from his metastatic melanoma. His prostate-specific antigen (PSA) was 30.8 ng/mL at the time of treatment initiation and responded rapidly to bicalutamide, reaching a nadir of 1.1 ng/mL in September 2018 (Fig 1A).

His melanoma was diagnosed from a shave biopsy of the right superior lateral lower back in August 2015 as localized ulcerated malignant melanoma, unclassified, with nevoid features. It was invasive to at least 1.45 mm and at least Clark level IV, and it had five mitoses/mm^2^. He subsequently had a microstaging excision and a right back excision with a negative sentinel lymph node in the right groin. In 2017, the patient noticed a new lump on his right lower back scar in 2017. In November 2017, excisional biopsies of his right lower back and right groin both showed metastatic melanoma, as confirmed by immunohistochemistry (IHC) showing positive staining for SOX-10 and MART-1. A 7-mm lesion was also detected on his posterior scalp and was also visible on magnetic resonance imaging of his brain and metabolically active on positron emission tomography (PET)/computed tomography (CT); hence, it was diagnosed as stage IV melanoma. Mutation testing showed an atypical N581I mutation in the *BRAF* gene, but wild-type status at the *BRAF* V600 codon. His PET/CT showed extensive osteosclerotic lesions, most of which were not fluorodeoxyglucose (FDG) avid, consistent with metastatic prostate cancer. The most avid lesion in the right ischium had a maximum standardized uptake value (SUV max) of 7.2 but was biopsied and found to be metastatic prostate adenocarcinoma, as confirmed by IHC assessment that demonstrated NKX3.1 expression. He began treatment of his metastatic melanoma with pembrolizumab 200 mg intravenous every 3 weeks and received four treatments before the pembrolizumab was stopped because of the development of pneumonitis. This treatment resulted in a complete response in his melanoma as assessed by physical examination of his scalp lesion. His pneumonitis was treated with a taper of prednisone, which was reduced to physiologic dosing by August 2018.

**FIG 2.**
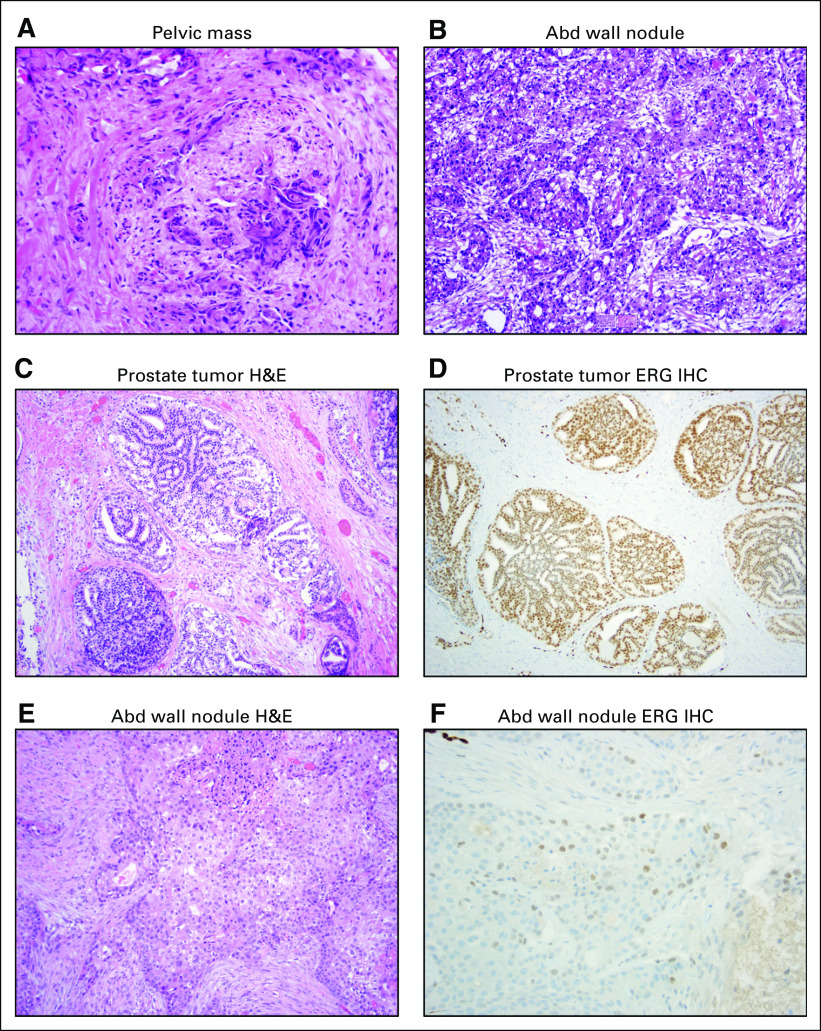
Histology and immunohistochemistry (IHC). (A) Hematoxylin and eosin (H&E) histology of pelvic mass biopsy showing squamous cell carcinoma. (B) H&E histology of the excisional biopsy of an abdominal (Abd) wall nodule, also showing squamous cell carcinoma. (C) H&E histology of the patient’s (untreated) prostate tumor at initial diagnosis showing prostate adenocarcinoma. (D) IHC for ERG of the primary prostate tumor showing strong labeling. (E) H&E histology of the Abd wall nodule biopsy specimen used for molecular analysis. (F) IHC for ERG of the Abd wall nodule showing much weaker labeling.

However, despite evidence of response in his melanoma by physical examination, there was concern for progression on PET/CT in June 2018, with FDG update in the descending colon/small bowel wall, perisplenic region, a mildly FDG-avid left internal iliac lymph node, and a right external iliac nodal conglomerate encasing the right ureter, 4.1 cm in diameter and with an SUV max of 11.7. By December 2018, the right pelvic mass had increased in size to 4.4 cm and in metabolic activity to SUV max 19.8 (Fig 1B). At this time, he was also noted to have multiple small lung nodules of unclear etiology. Because the appearance of the right pelvic mass was judged to be atypical for either prostate cancer or melanoma, it was biopsied by pelvic laparoscopy in November 2018 and was found on surgical pathology assessment to be consistent with a high-grade carcinoma with squamous features (Fig 2A). IHC assessment demonstrated neoplastic cells positive for p63 and GATA3, but negative for PSA and NKX3-1 expression. Urine cytology also showed atypical urothelial cells. We performed exome sequencing using the University of Michigan OncoSeq panel (MI-OncoSeq) and whole transcriptome analysis on formalin-fixed paraffin-embedded tissue from this biopsy. All patients enrolled in the MI-OncoSeq study provided written informed consent approved by the University of Michigan Institutional Review Board. Consent is inclusive of publishing information and/or images from participants (or their designate). However, results were limited because of low tumor content, estimated to be less than 10%. The variant allele fraction of mutations spanned 1% to 6%, and no copy number aberrations were detected (Table 1). Because the pelvic mass was causing pain and urinary obstruction, we elected to treat it with a platinum doublet active in urothelial carcinoma and carcinoma of unknown primary, despite not knowing the tissue of origin. In January 2019, we began carboplatin area under the curve 5 mg/mL/minute on day 1 and gemcitabine 1,000 mg/m^2^ on days 1 and 8 of 21-day cycles, and we completed six cycles before stopping because of progressive fatigue. The patient experienced some improvement in pain, but the size of the right pelvic mass was largely unchanged (Fig 1C).

**TABLE 1.**
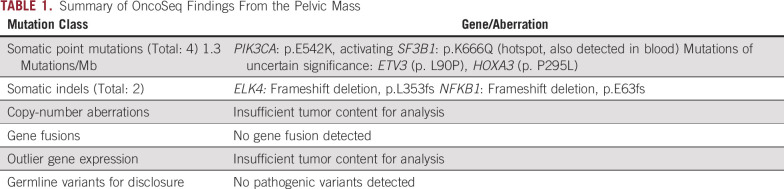
Summary of OncoSeq Findings From the Pelvic Mass

In June 2019, while being treated only with bicalutamide, the patient was noted on CT scan as having a 1.4-cm nodule in the superficial anterior abdominal wall, which subsequently became easily palpable and caused skin erythema. This nodule, together with smaller adjacent nodules, was removed by excisional biopsy in July 2019, where surgical pathology assessment demonstrated tumor features consistent with metastatic squamous cell carcinoma (Fig 2B). A fresh portion of this specimen was sent for analysis using the MI-OncoSeq platform. This specimen showed a much higher tumor content of 54%, allowing the discovery of additional molecular alterations.

### Precision Medicine Tumor Board Discussion

**FIG 3.**
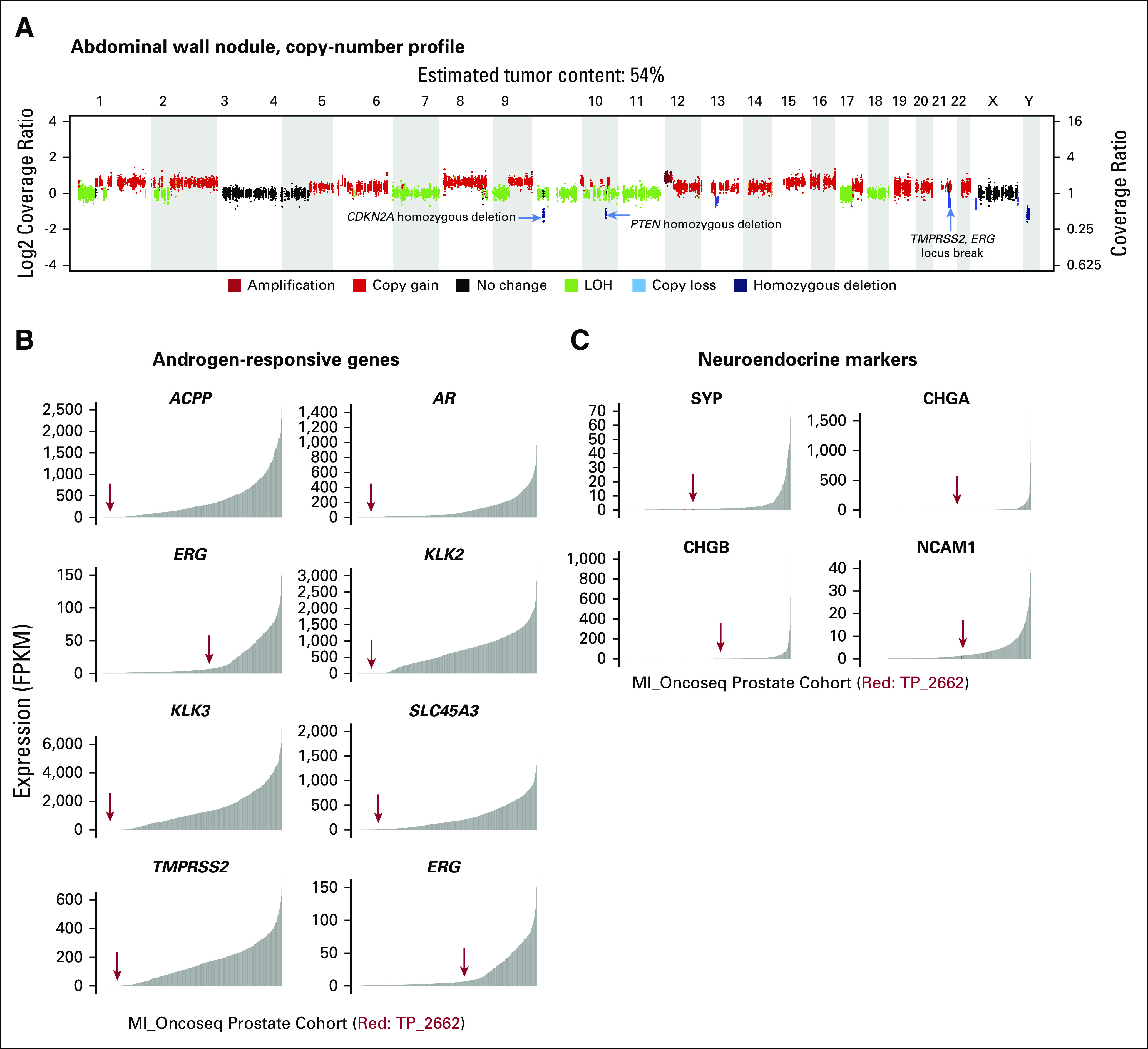
Next-generation sequencing. (A) Copy-number profile from the abdominal wall nodule highlighting the *TMPRSS2 ERG* locus break, a *PTEN* homozygous deletion, and a *CDKN2A* homozygous deletion. (B) Expression of androgen-responsive genes in the patient’s second sample OncoSeq transcriptomics data. (C) Expression of neuroendocrine marker genes in the patient’s second sample OncoSeq transcriptomics data. FPKM, fragments per kilobase of transcript per million mapped reads; LOH, loss of heterozygosity.

Results were discussed at the University of Michigan Precision Medicine Tumor Board in September 2019. Somatic aberrations detected in the previous sample were detected in the new biopsy specimen, suggesting clonal relatedness, and additional alterations consistent with the increased tumor content were noted as well (Table 2). Most curiously, we noted a few reads of chimeric transcripts supporting a gene fusion between TMPRSS2 exon 1 and ERG exon 2, accompanied by a focal deletion located in the intergenic region between the two genes and breakpoint visible on the copy number profile (Table 2 and Fig 3A). Fusions between the TMPRSS2 locus and Ets family transcription factors occur in nearly one half of prostate cancers in the United States and, to our knowledge, do not occur in other cancers.^1,2^ Given the presence of this pathognomonic gene fusion and the patient’s history of prostate adenocarcinoma, we concluded that his squamous cell carcinoma had arisen from his prostate cancer. To further confirm the findings, we subsequently performed IHC analysis, which showed positive ERG expression on the patient’s initial prostate transurethral resection, which was strongly positive for ERG protein (Figs 2C and 2D). We further performed ERG IHC assessment on the patient’s recent abdominal biopsy, which demonstrated focal and weak-to-moderate ERG protein expression, supporting a clonal phenotypic origin and evolution from the patient’s original conventional (acinar) prostatic adenocarcinoma (Figs 2E and 2F).

**TABLE 2.**
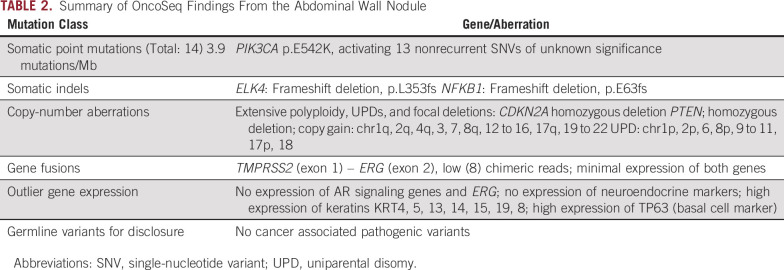
Summary of OncoSeq Findings From the Abdominal Wall Nodule

Armed with the knowledge that this patient’s squamous cell carcinoma was either a trans-differentiated or metaplastic variant of prostate cancer, we discussed the possible therapeutic implications at this point, when the patient was taking only single-agent bicalutamide. For prostate adenocarcinoma, the addition of medical castration, likely in addition to either abiraterone and prednisone or a nonsteroidal second-generation antiandrogen, would have been a reasonable next line of therapy. However, the transcriptomics data of the MI-OncoSeq platform showed low expression of the androgen receptor and androgen responsive genes *KLK2*, *KLK3* (PSA), *TMPRSS2*, *ACPP*, and *SLC45A3* (Fig 3B). In keeping with these findings, his PSA level was only 3.1 ng/mL at this point. Therefore, we concluded that additional therapy targeting androgens or the androgen receptor was unlikely to be successful. Similarly, we examined markers for neuroendocrine carcinoma, the most common nonadenocarcinoma type of prostate cancer. However, expression of the neuroendocrine markers *SYP*, *CHGA*, *CHGB*, and *NCAM1* were also low (Fig 3C). Therefore, we did not plan chemotherapy with a regimen such as carboplatin and etoposide, which is active against small-cell neuroendocrine carcinomas.

We examined the remainder of the molecular results in an effort to find alternative, clinically actionable molecular targets. We noted two alterations associated with PTEN and Akt signaling: an activating mutation in *PIK3CA* and a homozygous deletion in *PTEN* itself (Table 2). Inhibitors of mammalian target of rapamycin (mTOR) have been suggested for use in *PTEN*-deficient tumors. However, overall, the results for mTOR inhibitors in prostate cancer have been disappointing.^3^ Some randomized data support the use of PI3K inhibitors in metastatic castration-resistant prostate cancer.^4^ However, this was in combination with abiraterone, which made PI3K inhibitors less attractive in this case, given the patient’s near total lack of androgen signaling. Last, the homozygous deletion of *CDKN2B* could theoretically sensitize to CDK4/6 inhibitors. However, we decided against this option because CDK4/6 inhibitors have thus far shown disappointing results in prostate cancer.^5^ Therefore, we elected to treat with docetaxel, the most common cytotoxic chemotherapy drug for castration-resistant prostate cancer.

At this point, the patient’s lung nodules, which were previously indeterminate, had enlarged greatly (Fig 1D). We started treatment with docetaxel at 75 mg/m^2^ for one cycle, then decreased the dose to 60 mg/m^2^ because of fatigue, and completed three more cycles. We also attempted bicalutamide withdrawal for the adenocarcinoma component of his disease but resumed bicalutamide and added leuprolide a month later after his PSA continued to rise. The patient reported improvement in right groin pain shortly after initiation of docetaxel. CT completed after three cycles showed an interval decrease in the size of multiple bilateral lung nodules, and a decrease in size of his right pelvic mass, compatible with a therapeutic response (Fig 1E).

### Discussion

In this case, we used next-generation sequencing to determine that the patient’s squamous cell carcinoma was actually of prostate origin, because of the presence of a gene fusion between *TMPRSS2* and *ERG*. Transcriptomic and histologic analysis showed minimal evidence of androgen receptor signaling, but it also showed a lack of evidence of neuroendocrine differentiation. Such prostate cancers without evidence of androgen receptor signaling and without neuroendocrine markers have been termed “double-negative” prostate cancers.^6^ In more recent work profiling rapid autopsy specimens, investigators identified a squamous subtype of double-negative prostate cancer present in eight of 98 patients.^7^ Squamous histology prostate cancer was reported previously but was a rare finding before the development of advanced antiandrogens.^8^ However, the detection of squamous and other nonadenocarcinoma prostate cancer subtypes has become more common since the development of abiraterone and nonsteroidal second-generation antiandrogens,^6^ possibly as a means to escape continual selective pressure against androgen signaling, a phenomenon that had been described rarely in the past.^9^ Some of these double-negative prostate cancer (DNPC) tumors seem to be driven by fibroblast growth factor (FGF) alterations, and trials of FGF inhibitors have recently begun in advanced prostate cancer.^10^ Our patient did not have an FGF abnormality. Platinum-based chemotherapy is also commonly used to treat squamous cell neoplasms. In the current case, despite having squamous differentiation, this patient had only stable disease in response to platinum-based chemotherapy.

After using whole exome sequencing and RNA sequencing to identify this tumor as a squamous neoplasm of prostate origin, we elected to treat him with an agent approved for prostate cancer (docetaxel), an agent we would not have elected to use without knowing the tissue of origin. We elected not to pursue the therapies that could target molecular alterations in his tumor because of the known survival benefit of docetaxel in men with advanced prostate cancer, but therapies targeting his tumor’s molecular alterations remain options down the road if his disease progresses. In summary, this report demonstrates a case of transdifferentiation of a prostate adenocarcinoma to a DNPC tumor with squamous differentiation without evidence of an FGF alteration. Consideration should be given to docetaxel in patients with tumors of a similar phenotype.
